# Identification of large intergenic non-coding RNAs in bovine muscle using next-generation transcriptomic sequencing

**DOI:** 10.1186/1471-2164-15-499

**Published:** 2014-06-19

**Authors:** Coline Billerey, Mekki Boussaha, Diane Esquerré, Emmanuelle Rebours, Anis Djari, Cédric Meersseman, Christophe Klopp, Daniel Gautheret, Dominique Rocha

**Affiliations:** INRA, UMR1313, Unité Génétique Animale et Biologie Intégrative, Domaine de Vilvert, F-78352 Jouy-en-Josas, France; AgroParisTech, UMR1313, Unité Génétique Animale et Biologie Intégrative, Domaine de Vilvert, F-78352 Jouy-en-Josas, France; Institut de Génétique et Microbiologie, Université Paris-Sud, UMR8621, F-91405 Orsay, France; CNRS, UMR8621, Institut de Génétique et Microbiologie, F-91405 Orsay, France; INRA, UMR 444, Laboratoire de Génétique Cellulaire, INRA Auzeville, BP 52627, F-31326 Castanet-Tolosan Cedex, France; GeT-PlaGe, Genotoul, INRA Auzeville, BP 52627, F-31362 Castanet-Tolosan Cedex, France; INRA, SIGENAE, UR 875, INRA Auzeville, BP 52627, F-31326 Castanet-Tolosan Cedex, France

**Keywords:** Cattle, Muscle, RNA-Seq, Beef, Long non-coding RNA

## Abstract

**Background:**

The advent of large-scale gene expression technologies has helped to reveal in eukaryotic cells, the existence of thousands of non-coding transcripts, whose function and significance remain mostly poorly understood. Among these non-coding transcripts, long non-coding RNAs (lncRNAs) are the least well-studied but are emerging as key regulators of diverse cellular processes. In the present study, we performed a survey in bovine *Longissimus thoraci* of lincRNAs (long intergenic non-coding RNAs not overlapping protein-coding transcripts). To our knowledge, this represents the first such study in bovine muscle.

**Results:**

To identify lincRNAs, we used paired-end RNA sequencing (RNA-Seq) to explore the transcriptomes of *Longissimus thoraci* from nine Limousin bull calves. Approximately 14–45 million paired-end reads were obtained per library. A total of 30,548 different transcripts were identified. Using a computational pipeline, we defined a stringent set of 584 different lincRNAs with 418 lincRNAs found in all nine muscle samples. Bovine lincRNAs share characteristics seen in their mammalian counterparts: relatively short transcript and gene lengths, low exon number and significantly lower expression, compared to protein-encoding genes. As for the first time, our study identified lincRNAs from nine different samples from the same tissue, it is possible to analyse the inter-individual variability of the gene expression level of the identified lincRNAs. Interestingly, there was a significant difference when we compared the expression variation of the 418 lincRNAs with the 10,775 known selected protein-encoding genes found in all muscle samples. In addition, we found 2,083 pairs of lincRNA/protein-encoding genes showing a highly significant correlated expression. Fourteen lincRNAs were selected and 13 were validated by RT-PCR. Some of the lincRNAs expressed in muscle are located within quantitative trait loci for meat quality traits.

**Conclusions:**

Our study provides a glimpse into the lincRNA content of bovine muscle and will facilitate future experimental studies to unravel the function of these molecules. It may prove useful to elucidate their effect on mechanisms underlying the genetic variability of meat quality traits. This catalog will complement the list of lincRNAs already discovered in cattle and therefore will help to better annotate the bovine genome.

**Electronic supplementary material:**

The online version of this article (doi:10.1186/1471-2164-15-499) contains supplementary material, which is available to authorized users.

## Background

Over the past decade, genome-wide transcriptional studies discovered that a large fraction of the eukaryotic genomes is transcribed in a heterogeneous population of noncoding RNAs (ncRNAs). These are transcripts that are not translated into a protein but act as functional RNAs. ncRNAs shorter than 200 nucleotides are usually identified as small/short ncRNA and include PIWI-interacting RNAs (piRNAs), endogeneous small interfering RNAs (siRNAs) and microRNAs (miRNAs) but also classical ncRNAs, such as ribosomal RNAs (rRNAs), transfer RNAs (tRNAs) and small nucleolar RNAs (snoRNAs); whereas those longer than 200 nucleotides are classified as long ncRNAs (lncRNAs). LncRNAs can be classified as lincRNAs (long intergenic non-coding RNAs) that are transcribed adjacent to protein-coding genes, eRNAs (enhancer RNAs that are transcribed within the enhancer regions), intronic lncRNAs (transcribed within the introns of protein-coding genes) and antisense lncRNAs (transcribed from the opposite genomic strand relative to protein-coding genes) [1, 2]. In the past few years, an increasing number of lncRNAs have been discovered in eukaryotic organisms, ranging from nematodes to humans [3–17]. For example, the most recent report of the ENCODE (Encyclopedia of DNA Elements) project published in September 2012, described 9,640 lncRNA loci in comparison to 20,687 protein-coding genes in 15 human cell lines [14–16].

LncRNAs can be polyadenylated or non-polyadenylated [17, 18], spliced or mono-exonic unspliced and the expression level of individual lncRNAs is generally lower than the level of expression of the typical protein-coding mRNAs [17, 19–22], and some lncRNAs have high tissue specificity [21–24].

Despite the fact that only few lncRNAs have been characterized experimentally in detail to date, it is already known that they can act via diverse mechanisms [25] and can play regulatory and structural roles in almost every important biological process, such as X-chromosome inactivation and genomic imprinting, nuclear compartmentalization and architecture, cell fate specification, RNA splicing, translational control, and chromatin modification [26].

Because of the key role of lncRNAs in regulation of gene expression and therefore possible impact on phenotypes, it is important to identify most lncRNAs. Catalogues of lncRNA have been established for many species, including cattle [27–29]. For example, Huang *et al*. (2012) have identified a total of 449 putative lncRNAs located in 405 intergenic regions using public bovine-specific expressed sequence tags sequences [28]. More recently, Weikard *et al.* (2013) predicted more than 4,000 potential lncRNAs in bovine skin using RNA-Seq data [29]. The current number of bovine lncRNAs identified is rather low compared to more than 9,000 lncRNAs found in human, suggesting that more efforts are needed to discover all bovine lncRNAs.

In the present study, we identified lncRNAs in bovine *Longissimus thoraci*, using a whole-transcriptome sequencing approach. To our knowledge, this represents the first study done in bovine muscle. For this purpose, muscle samples from nine different Limousin bulls were analysed. We have identified more than 500 different lincRNAs and 13 out of the 14 selected lincRNAs were validated experimentally. The RNA-Seq data and the collection of newly discovered lincRNAs improve the genomic resources available for cattle, especially for beef breeds. This collection of lincRNAs may prove useful to study their link with genetic variability of meat quality traits.

## Results and discussion

### RNA sequencing and assembly of a muscle transcriptome

To identify lincRNAs expressed in the bovine *Longissimus thoraci*, we used paired-end RNA sequencing (RNA-Seq) from nine Limousin bull calves. We used already published data from three Limousin animals [30] and poly(A)-enriched mRNA from six new Limousin bull calves were retrotranscribed and subjected to high-throughput sequencing. The six RNA-Seq libraries were barcode-tagged and sequenced on two lanes (3 libraries per lane) of an Illumina HiSeq2000 sequencer. The reads were then de-multiplexed to assign reads to each sequenced sample according to its barcode index.

Sequencing of all nine cDNA libraries generated a total of 300,941,530 raw paired-end reads with a length of 100 bases, resulting in a total of 60 gigabases. Approximately 14 to 45 million paired-end reads were obtained for each library. The reads were then aligned using TopHat [31] onto the bovine UMD3.1 reference genome sequence. 65% to 75% of the reads were aligned onto the bovine genome, and 82% to 92% of the mapped reads were aligned properly paired (Table 1). Transcripts were reconstructed using Cufflinks [32], resulting in the assembly of a total number of 131,753 transcripts (30,548 different genes) with at least one paired-end read.

**Table 1:** **Summary of reads mapping to the bovine transcriptomes**

	LIM1	LIM2	LIM3	LIM4	LIM5	LIM6	LIM7	LIM8	LIM9
Number of reads	86,352,760	72,251,962	90,678,870	74,649,210	72,416,218	80,220,062	38,198,732	27,278,276	59,836,970
Number of bases (in Gb)	8.64	7.23	9.07	7.46	7.24	8.02	3.82	2.73	5.98
Number of mapped reads	65,739,933	54,576,643	68,916,620	61,346,058	50,206,044	61,543,271	30,174,318	21,840,568	46,205,282
% mapped reads	76.13	75.54	76	82.18	69.33	76.72	78.99	80.07	77.22
Number of uniquely mapped reads	61,587,716	51,574,445	65,139,874	56,117,512	47,169,853	58,093,383	28,421,388	20,558,269	43,701,020
% uniquely mapped reads	70.82	71.38	71.84	75.17	65.14	72.42	74.4	75.36	73.03
Number of uniquely mapped paired-reads	32,792,300	26,484,662	32,399,058	25,646,394	24,089,814	30,477,552	15,891,664	12,590,540	23,843,178
% uniquely mapped paired-reads	86.22	84.15	82.29	90.86	85.68	87.09	82.47	92.04	86.62

Similar RNA-Seq read mapping rates were obtained in other RNA-Seq bovine studies [33–38]. For example, Baldwin and collaborators found by sequencing the rumen epithelium that ~71% of the reads uniquely mapped to specific regions of the bovine genome [36]. Interestingly a comparable number of genes has been detected in bovine skin in a RNA-Seq project using a similar sequencing coverage and bioinformatics pipeline [29].

Raw gene expression levels were estimated by measuring the normalised count number for each transcript (number of reads per transcript divided by the total number of mapped reads, for each sample). The five most frequent transcripts are shown in Table 2. These five genes (actin alpha skeletal muscle, myosin 1 and myosin 2, nebulin, titin) represent nearly 20% of all sequencing reads mapped to the bovine genome and are all associated with muscle structure. These results were consistent with the physiological role of genes expected in the surveyed tissue.

**Table 2:** **Top five transcripts with most assigned reads**

Locus	Gene name	Gene symbol	Number of reads (9 samples)	% total number of reads
XLOC_016519	titin	TTN	8,065,194	6.07
XLOC_015363	myosin 1	MYH1	5,127,160	3.86
XLOC_016610	nebulin	NEB	4,195,679	3.16
XLOC_015364	myosin 2	MYH2	3,787,472	2.85
XLOC_025531	actin, alpha skeletal muscle	ACTA1	3,549,948	2.67

Not all genes were expressed among the nine selected samples. Transcripts corresponding to 20,907 different genes were detected in all nine samples, while approximately 32% of the genes were expressed in only some of the samples, including 1,443 different genes (~5%) only expressed in one sample.

Transcript models predicted with Cufflinks for each sample were sorted into different categories using the bovine genome annotation (Table 3). Approximately between 57-66% (mean +/− SD: 61% +/− 3%) of the transcripts correspond to already known transcripts. Surprisingly, between ~18-22% (mean +/− SD: 20% +/− 1%) of the transcript models generated correspond to novel isoforms of known genes presumably from alternative splicing events (“j” class). This indicates that the bovine genome remains poorly annotated and that a large number of new transcript isoforms are still to be described. Interestingly, more than 2-4% (mean +/− SD: 3% +/− 1%) of the transcript models are predicted as unknown intergenic transcripts (“u” class). It is notable that some transcripts are classified into different categories. For example, a transcript model could be part of an already known transcript and at the same time it could be part of the novel isoform category, as the RNA-Seq data indicates a novel exon.

**Table 3:** **Number of mapped sequencing reads for each different class of assembled transcripts**

	LIM1	LIM2	LIM3	LIM4	LIM5	LIM6	LIM7	LIM8	LIM9
**Code**									
**e**	841	898	884	711	904	757	655	547	741
**=**	26,052	26,029	26,072	26,075	26,237	26,064	2,6158	26,139	2,6117
**x**	285	276	250	227	278	271	195	177	247
**s**	2	2	4	2	5	3	3	2	3
**j**	9,262	9,660	9,476	9,008	7,811	9,121	8,111	8,223	8,753
**c**	2	2	2	2	2	3	3	2	3
**p**	714	629	678	693	749	711	529	670	615
**u**	1,459	1,510	1,988	1,123	1,842	1,462	868	1,058	985
.	3,577	3,411	3,585	3,274	3,690	3,674	2,344	2,079	3,296
**o**	413	434	448	377	354	427	317	288	362
**i**	1,611	1,701	2,271	1,316	2,369	1,725	967	603	1,446

**e**, single exon overlapping a reference exon and at least 10 bp of a reference intron indicating a possible pre-mRNA fragment; =, complete match of intron chain; **x**, exonic overlap with reference on the opposite strand; **s**, an intron of the transfrag overlaps a reference intron on the opposite strand (likely due to read mapping errors); **j**, potentially novel isoform; **c**, contained in reference; **p**, possible polymerase run-on fragment (within 2 kb of a reference transcript); **u**, unknown intergenic transcript; ., tracking file only, indicates multiple classifications; **o**, generic exonic overlap with a reference transcript; **i**, a single exon transcript falling entirely within a reference intron.

### Identification of putative lincRNAs

To identify lncRNAs, we developed a stringent filtering pipeline to discard transcripts with evidence for protein-coding potential. We identify putative lncRNAs by considering their open reading frame, their phylogenetic conservation across species and homology with known proteins and protein domains. The reads were generated from non-directional RNA-Seq libraries, we therefore focus our effort on unknown intergenic transcripts and therefore could only identify putative lincRNAs.

First, a minimal transcript size criterion was applied. Transcripts with multiple exons and larger than 200 nt were used. This analysis resulted in the identification of 2,291 putative multi-exonic intergenic transcripts (1,127 different loci).

Second, we used PhyloCSF to score the coding potential of unknown multi-exonic transcripts using multi-species alignments. PhyloCSF scores were calculated for the 2,291 putative multi-exonic intergenic transcripts and two control sets, one of 10,000 known protein-coding genes found in our RNA-Seq libraries and one with 438 already known bovine lncRNAs [28]. We set the PhyloCSF threshold empirically to a value retaining 73% of the known bovine ncRNAs while removing 7% of protein-coding transcripts. This filter retained 1,383 putative non-coding transcripts (798 different loci).

Third, we used CPAT on the same 2,291 putative multi-exonic intergenic transcripts, in order to assess their coding potential with a second prediction method. To determine the optimum cut-off value, CPAT was trained using a set of 10,000 bovine known protein-encoding transcripts and a set of 10,000 bovine non-coding sequences and a 10-fold cross-validation analysis was performed to estimate the prediction accuracy. A cut-off value of 0.348 was selected, maximising specificity and sensitivity (98.4%) (Additional file 1: Figure S1). This procedure identified 2,085 transcripts (1,060 different loci) as potential non-coding RNAs. The intersection of PhyloCSF and CPAT predicted 1,330 transcripts (773 different loci) as potential non-coding RNAs.

Finally, we removed any remaining transcripts of uncertain coding potential that had similarity to known proteins or protein domains recorded in the Pfam database. The resulting set contained 1,277 transcript models corresponding to 584 different putative non-coding genes (Additional file 2: Table S1). There is a possibility that some real lincRNAs would have not been detected because of our stringent selection criteria. For example, some real lincRNAs could be lost owing to the chosen minimum transcript size. We might also have missed some lowly expressed lincRNA genes due to our moderate sequencing depth.

Comparison of the genomic position of the 584 different genes encoding putative lincRNAs found in bovine *Longissimus thoraci* with mapping positions of previously identified bovine lncRNAs publicly available in the NONCODE database (release 4) [39] show that 163 (~28%) of our lincRNAs overlap with previously described bovine non-coding RNA genes (Additional file 3: Table S2).

### Characterisation of identified lincRNAs

The chromosomal location of the genes encoding these 584 putative lincRNAs is presented in the Additional file 4: Figure S2. The chromosomal distribution usually reflects the gene content of the chromosomes: larger chromosomes have more lincRNA loci than shorter chromosomes. For example, we found 35 genes encoding putative lincRNAs on BTA10, whereas only 10 on BTAX.

Previous studies have shown that genes encoding lncRNAs are shorter in length, have shorter transcripts and have fewer exons than protein-coding genes [6, 11, 40]. To determine whether the bovine muscle lincRNAs we detected have the same features, we compared the size of 584 lincRNA genes to 15,358 protein-encoding genes detected in our RNA-Seq data. The lincRNAs represent much shorter gene length on average than protein-encoding genes (33.13 +/− 52.66 kb versus 45.63 +/− 78.65 kb, *P* < 10^−8^, Student’s’s *t-*test). We compared also the size of the 4,496 transcripts corresponding to the 584 lincRNA genes to the size of 97,172 transcripts of the selected protein-encoding genes. The lincRNAs have shorter transcript on average than protein-encoding genes (3.12 +/− 2.42 kb versus 3.65 +/− 2.85 kb, *P* < 10^−45^, Student’s *t-*test). In addition, lincRNA genes show also fewer exons than protein-encoding genes (3.04 +/− 2.0 versus 10.43 +/− 10.50, *P* = 0, Student’s test).

To determine whether the bovine muscle lincRNAs we detected have the same expression feature, we compared the normalised quantified expression levels of the 584 lincRNAs to that of the 15,358 known selected protein-encoding genes. Our comparison indicates that the identified bovine lincRNAs do show significant lower expression than the protein-encoding genes (4.16 × 10^−5^ +/− 3.70 × 10^−4^ versus 5.62 × 10^−5^,+/− 8.44 × 10^−4^, *P* < 10^−3^, Student’s *t-*test). Previous studies also showed that lincRNAs are expressed at significantly lower levels than are protein-coding transcripts [6, 11, 40].

### LincRNA gene expression

Not all 584 lincRNA genes were expressed among the nine selected samples. 418 genes were detected in all samples, while 55 genes were detected in less than five samples (Additional file 2: Table S1). The highest number of putative lincRNAs (572 or 97% of all predicted lincRNAs) was found in sample LIM1.

The sequencing reads derived from the 584 different putative lincRNA made up a bit more than 1% of all the paired-end reads mapped onto the bovine genome. Three genes encoding putative lincRNAs had each more than 0.05% of the total mapped paired-end sequencing reads. The gene encoding a putative lincRNA with the most sequencing reads (XLOC_026244) was represented by 0.28% of the total sequencing reads. These lincRNAs with a relatively high expression level in bovine *Longissimus thoraci* might play an important role in muscle function.

As our study identified lincRNAs from different samples but from the same tissue, it is possible to analyse the inter-individual variability of the gene expression level of the identified lincRNAs. We calculated the coefficient of variation for each lincRNA, from the expression level measured with the nine different *Longissimus thoraci* samples and compared it with the gene expression variation of the selected protein-coding genes. Interestingly, there was a significant difference when we compared the expression variation of the 418 lincRNAs with the 10,775 known selected protein-encoding genes found in all nine muscle samples (46.67 +/− 30.12 versus 36.58 +/− 22.69, *P* < 10^−11^, Student’s *t-*test). The higher averaged expression variation of the identified lincRNAs suggests a loosened gene expression regulation of these genes compared to protein-encoding genes. To our knowledge, it is the first time that this is described.

The observed higher gene expression variation of lincRNA genes could be due to a higher number of polymorphisms within the regulatory regions. To support this hypothesis we compared the SNP density (number of SNPs per kb) of the regulatory region of the lincRNA genes and protein-coding genes. We mapped SNPs from Ensembl (Ensembl Variation version 74) to the upstream (−10 kb to 0 bp from the predicted transcription starting site) and downstream (up to 1 kb after the stop codon) regions of the lincRNA genes and protein-encoding genes. There was no significant difference when we compared the averaged SNP density in the downstream regions of the 418 lincRNAs with the one of the 15,358 known selected protein-encoding genes (7.16 × 10^−3^ +/− 4.66 × 10^−3^ versus 7*.*09 × 10^−3^ +/− 4.41 × 10^−3^, *P* = 0.7%, Student’s *t-*test). In addition, there was no significant difference when we compared the averaged SNP density in the upstream regions of the group of genes (7.66 × 10^−4^ +/− 6.34 × 10^−4^ versus 7*.*20 × 10^−4^ +/− 5.84 × 10^−4^, *P* = 0.54%, Student’s *t-*test). The lack of difference in SNP densities do not rule out that the observed higher gene expression variation of lincRNA genes could be due to the effect of polymorphisms within the regulatory regions. However, the SNP densities should be determined after sequencing the whole-genome or the regulatory region of the lincRNA genes and protein-coding genes of the nine animals for which the expression data was generated.

The expression variation of the identified genes might be affected by the moderate sequencing depth obtained for some samples. More work is therefore required to confirm the higher averaged expression variation seen with lincRNA genes compared to protein-coding genes.

### Co-expression analysis

We have in our study the expression levels of the identified lincRNAs and of known protein-encoding genes in nine samples from the same tissue. It is therefore possible to analyse the co-expression of lincRNAs with protein-encoding genes. Using the normalised expression levels, we calculated the Spearman's rank correlation coefficient for each lincRNA with each protein-encoding gene. We found after correction for multiple testing 2,081 pairs of lincRNA/protein-encoding genes showing a highly correlated expression (*P* < 1.11 × 10^−8^) (Additional file 5: Table S3). 45 different lincRNA genes and 966 different protein-encoding genes had their expression highly correlated. 14 lincRNAs showed correlations with more than 20 protein-encoding genes. Four lincRNA genes (XLOC_009350, XLOC_018437, XLOC_021729 and XLOC_024598) had the most correlations, with 192 different protein-encoding genes. All lincRNA/protein-encoding gene pairs showing highly correlated expression had the same correlation and *P*-values (*rho* = 1, *P* < 0). No anti-correlated lincRNA/protein-coding gene pairs were found. There were neither no pairs of lincRNA/protein-encoding genes located at less than 2 Mb apart, suggesting a lack of *cis*-regulation among the paired genes we detected in bovine muscle. However, we found 88,888 co-expression correlations without the Bonferroni correction for multiple testing (*P* < =0.05) between 95 different lincRNAs and 15,209 different protein-encoding genes, including 1,738 *cis* and 33,896 anti- correlations.

### Validation of novel lincRNAs

**Figure 1:**
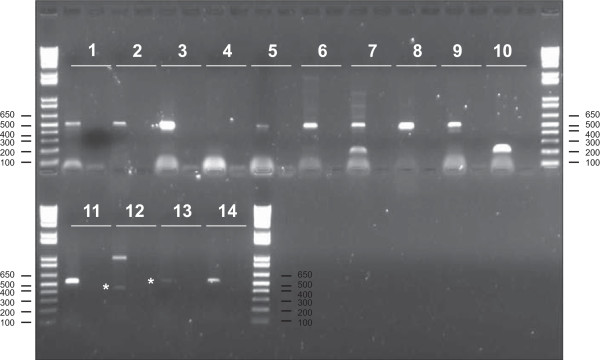
**Validation of selected lincRNAs using RT-PCR.** Selected lincRNAs are numbered from 1 to 14 as detailed in Additional file 6: Table S4. The first and second lanes are with PCR products using cDNA or without cDNA (negative control), respectively. *indicates faint but specific amplification products.

To confirm that the identified bovine lincRNAs are transcribed *in vivo*, 14 lincRNAs detected in all 9 samples were randomly selected for RT-PCR validation. 13 out of the 14 selected lincRNAs could be amplified using total RNA from *Longissimus thoraci*, as shown in Figure 1. All amplification products have the expected size; however for lincRNA XLOC_021462 we obtain an extra band of ~1,000 bp. The high percentage of validation suggests that most putative lincRNAs might be truly expressed *in vivo*.

### Functional lincRNA candidates

The positions of the 584 genes encoding putative linRNAs were compared to the position on the UMD3.1 bovine genome assembly of know quantitative trait loci (QTLs) deposited in the public database AnimalQTLdb [41]. 556 loci were located in 2,389 different QTL regions, including 507 lincRNAs within 550 QTLs for meat quality/muscle-related traits (Additional file 6: Table S4). For example, 110 different putative lincRNAs are found in 48 QTL regions for meat tenderness; whereas 281 putative lincRNAs are within 86 QTLs for marbling score. QTLs were sorted into two groups (meat quality/muscle-related QTLs *versus* other QTLs) and the number of lincRNAs found in these two groups were counted. We then performed a Chi-squared test and found a significant difference (*P* = 8.63 × 10^−63^) in the number of lincRNAs between the two groups (Additional file 7: Table S5), suggesting an enrichment of SNPs in meat/muscle related QTLs. The high number of putative lincRNAs located within known QTL regions, particularly in chromosomal regions harbouring QTLs for meat quality-related traits, indicates that the collection of lincRNAs found in the *Longissimus thoraci* transcriptome may prove useful to elucidate their effect on mechanisms underlying the genetic variability of meat quality traits.

## Conclusions

The present study represents the first analysis of large intergenic non-coding genes discovered in bovine muscle. Using a computational pipeline that we developed to analyse RNA-Seq data, we identified 584 different novel putative lincRNAs. We could validate by RT-PCR 13 out of fourteen selected putative lincRNAs, suggesting that most putative lincRNAs might be truly expressed *in vivo*. The identified putative bovine lincRNA genes share most features with mammalian counterparts.

As our study identified lincRNAs in *Longissimus thoraci* from nine different Limousin animals, we could analyse the variability of the gene expression level of the identified lincRNAs and compared it with the gene expression variation of the known selected protein-coding genes. We found a higher averaged expression variation for the identified lincRNAs suggesting a loosened gene expression regulation. We also analysed the co-expression of lincRNAs with protein-encoding genes and found 2,093 pairs of lincRNA/protein-encoding genes showing a highly correlated expression.

Some of the lincRNAs expressed in muscle are located within quantitative trait loci for meat quality traits. Future experimental studies are required to unravel the function of these molecules and to elucidate their effect on mechanisms underlying the genetic variability of meat quality traits.

The lincRNAs identified here will complement the catalog of lincRNAs already discovered in cattle and therefore will help to better annotate the bovine genome.

## Methods

### Animal ethics

All animal experimentation complied with the French Veterinary Authorities’ rules. No ethics approval was required by a specific committee, as the selected animals were not animals bred for experimental reasons.

### Animals and tissue samples

The study was conducted with nine Limousin bull calves from a large study on the genetic determinism of beef and meat quality traits [42]. The nine bull calves were not closely related to one another (for at least 4 generations) were fattened in a single feedlot and fed *ad libidum* with wet corn silage. They were humanely slaughtered in an accredited commercial slaughterhouse when they reached 16 months. *Longissimus thoraci* (LT) muscle samples were dissected immediately after death and tissue samples were snap frozen in liquid nitrogen and stored at −80°C until analysis.

### RNA isolation and sequencing

RNA extraction and sequencing were performed as previously described [30]. Briefly, after transfer to ice-cold RNeasy RLT lysis buffer (Qiagen, Courtaboeuf, France), LT tissue samples were homogenised using a Precellys tissue homogeniser (Bertin Technologie, Montigny-le-Bretonneux, France). Total RNA was isolated using RNeasy Midi columns (Qiagen) and then treated with RNAse-free DNase I (Qiagen) for 15 min at room temperature according to the manufacturer’s protocols. The concentration of total RNA was measured with a Nanodrop ND-100 instrument (Thermo Scientific, Ilkirch, France) and the quality was assessed with an RNA 6000 Nano Labchip kit using an Agilent 2100 Bioanalyzer (Agilent Technologies, Massy, France). All nine samples had an RNA Integrity Number (RIN) value greater than eight.

The mRNA-Seq libraries were prepared using the TruSeq RNA Sample Preparation Kit (Illumina, San Diego, CA) according to the manufacturer's instructions. Briefly, Poly-A containing mRNA molecules were purified from 4 μg total RNA of each sample using oligo(dT) magnetic beads and fragmented into 150–400 bp pieces using divalent cations at 94°C for 8 min. The cleaved mRNA fragments were converted to double-stranded cDNA using SuperScript II reverse transcriptase (Life Technologies, Saint Aubin, France) and primed by random primers. The resulting cDNA was purified using Agencourt AMPure® XP beads (Beckman Coulter, Villepinte, France). Then, cDNA was subjected to end-repair and phosphorylation and subsequent purification was performed using Agencourt AMPure® XP beads (Beckman Coulter). These repaired cDNA fragments were 3′-adenylated producing cDNA fragments with a single ‘A’ base overhung at their 3′-ends for subsequent adapter-ligation. Illumina adapters containing indexing tags were ligated to the ends of these 3′-adenylated cDNA fragments followed by two purification steps using Agencourt AMPure® XP beads (Beckman Coulter). Ten rounds of PCR amplification were performed to enrich the adapter-modified cDNA library using primers complementary to the ends of the adapters. The PCR products were purified using Agencourt AMPure® XP beads (Beckman Coulter) and size-selected (200 ± 25 bp) on a 2% agarose Invitrogen E-Gel (Thermo Scientific). Libraries were then checked on an Agilent Technologies 2100 Bioanalyzer using the Agilent High Sensitivity DNA Kit and quantified by quantitative PCR with the QPCR NGS Library Quantification kit (Agilent Technologies). After quantification, tagged cDNA libraries were pooled in equal ratios and a final qPCR check was performed post-pooling. The pooled libraries were used for 2 × 100 bp paired-end sequencing on one lane of the Illumina HiSeq2000 with a TruSeq SBS v3-HS Kit (Illumina). After sequencing, the samples were demultiplexed and the indexed adapter sequences were trimmed using the CASAVA v1.8.2 software (Illumina).

### Transcriptome assembly and gene expression counts

RNA-Seq reads from each sample were aligned to the UMD3.1 *Bos taurus* reference genome [43, 44] with TopHat (version 1.4.0) using the default settings and a maximum intron size of 50,000 bp and the expected mean inner distance between paired-reads of 300 bp (−I 50000 –r 300) [31]. Only uniquely mapped and properly paired reads were then assembled with Cufflinks [32]**(**version 2.0) and using Ensembl’s bovine gene annotation (version 71). A unique set of all transcripts found among the nine samples was generated using Cuffcompare and all assembled transcripts were quantified in each sample using HTSeq-count ([45], version 0.5.4).

### Analysis of coding potential

Classification of each transcript as either coding or noncoding was determined using a step-wise pipeline.

First, all candidate transcript models were scored with PhyloCSF [46] to determine their coding potential. PhyloCSF uses a multispecies nucleotide sequence alignment to identify conserved protein-coding region, based on a statistical comparison of phylogenetic codon models. We used a five-species alignment between cow, human (hg19), mouse (mm9), rat (rn4) and dog (CanFam2). Pairwise alignments were obtained from the UCSC website (http://hgdownload.soe.ucsc.edu/downloads.html#cow). All transcripts with a negative score were retained as potential non-coding candidates.

Second, the Coding Potential Assessment Tool (CPAT) [47] was applied (version 1.2.1) on all candidate transcript models in order to assess their coding potential by a second independent prediction method. According to the authors [47] the CPAT coding probability score ranges between 0 and 1, and the optimum cut-off value for protein coding probability varies depending on the species to be analysed. To determine the cut-off value, CPAT was trained using a set of 10,000 bovine known protein-encoding transcripts and a set of 10,000 bovine non-coding sequences. The set of non-coding sequences included 3,801 bovine short non-coding genes and 6,199 bovine intronic sequences (larger than 200 bases). Bovine known protein-encoding transcripts and intronic sequences were extracted randomly from Ensembl using the bovine gene annotation (version 71). The two training sets were randomly split into ten different parts to perform a 10-fold cross-validation analysis. CPAT was trained on one part and the predictions were made on the remaining nine parts. This process was repeated ten times, so each sample was used once for the prediction. Prediction accuracy (sensitivity and specificity) was obtained for each repetition. Sensitivity and specificity were calculated as follows: Sensitivity = TP/(TP + FN) and Specificity = TN/(TN + FP). Where TP, FP, TN and FN are the numbers of true positives (non-coding sequences predicted to be non-coding), false positives (protein-coding transcripts predicted to be non-coding), true negatives (protein-coding transcripts predicted to be coding) and false negatives (non-coding sequences predicted to be coding). The cut-off value was selected to maximize specificity and sensitivity. In order to extract potential non-coding transcripts with a high reliability from our dataset, all transcripts with a score below 0.348 were retained as potential non-coding RNAs.

Finally, all candidate transcripts were translated *in silico* into the three possible open reading frames using a custom script and compared against the Pfam protein families database ([48], version 27.0) with the hmmscan algorithm (package HMMER3, version 3.1b1). Candidate transcript models with known protein motifs were discarded.

### Validation by RT-PCR

The RT-PCR primers were designed using Primer3 (http://bioinfo.ut.ee/primer3/) with the optimal PCR product length set between 191 and 513 bp. Primer sequences are presented in Additional file 8: Table S6. The PCR primers were synthesised by Eurofins MWG Operon. Each PCR primer pair was tested using a pool of cDNA made of two animals.

One microgram of DNase I-treated total RNA was used to synthesize the first strand of cDNA using the SuperScript First-Strand Synthesis System III for RT-PCR (Invitrogen) according to the manufacturer’s instructions and applying a combination of 50 ng random hexamers.

Polymerase chain reactions were performed in 25 μl using 60 ng cDNA, 1× PCR buffer, 1.5 mm MgCl_2_, 0.2 mm of each dNTP, 0.3 μM of each primer and 1U Go*Taq* DNA polymerase (Promega). The following touchdown cycling protocol was used: 95°C for 2 min, followed by 13 cycles of 95°C for 1 min, 1 min of annealing (the annealing temperature was progressively lowered from 68 to 56°C in steps of 1°C every cycle) and 72°C for 1 min. These initial cycles were followed by 20 cycles of 95°C for 1 min, 55°C for 1 min and 72°C for 1 min, and a final extension step at 72°C for 2 min. 10 μl of each PCR product was then analysed by gel electrophoresis with a 1% agarose gel.

### Statistical analysis

Spearman's rank correlation coefficient were calculated for the correlation studies using the statistical *R* package.

### Data availability

The sequencing data have been submitted to the European Nucleotide Archive (accession numbers ERP002220 and E-MTAB-2646).

## Electronic supplementary material

Additional file 1: Figure S1: Performance evaluation using 10-fold cross-validation. (PPTX 75 KB)

Additional file 2: Table S1: List of candidate lincRNA genes. (XLSX 36 KB)

Additional file 3: Table S2: List of lincRNA genes previously identified in cattle. (XLSX 58 KB)

Additional file 4: Figure S2: Distribution of lincRNA genes over all bovine chromosomes. (DOCX 12 KB)

Additional file 5: Table S3: List of lincRNA/protein-encoding gene pairs with highly correlated expression. (XLSX 158 KB)

Additional file 6: Table S4: List of putative lincRNAs located within known QTL regions. (XLSX 5 MB)

Additional file 7: Table S5: Chi-squared test details. (XLSX 4 MB)

Additional file 8: Table S6: Primer sequences used for RT-PCR. (DOCX 12 KB)
