# Supplementary figures and images for: Identification of large intergenic non-coding RNAs in bovine muscle using next-generation transcriptomic sequencing
Source: BMC Genomics. 2014 Jun 19;15(1):499. doi: 10.1186/1471-2164-15-499 (PMC4073507; doi:10.1186/1471-2164-15-499)

## Slide 1
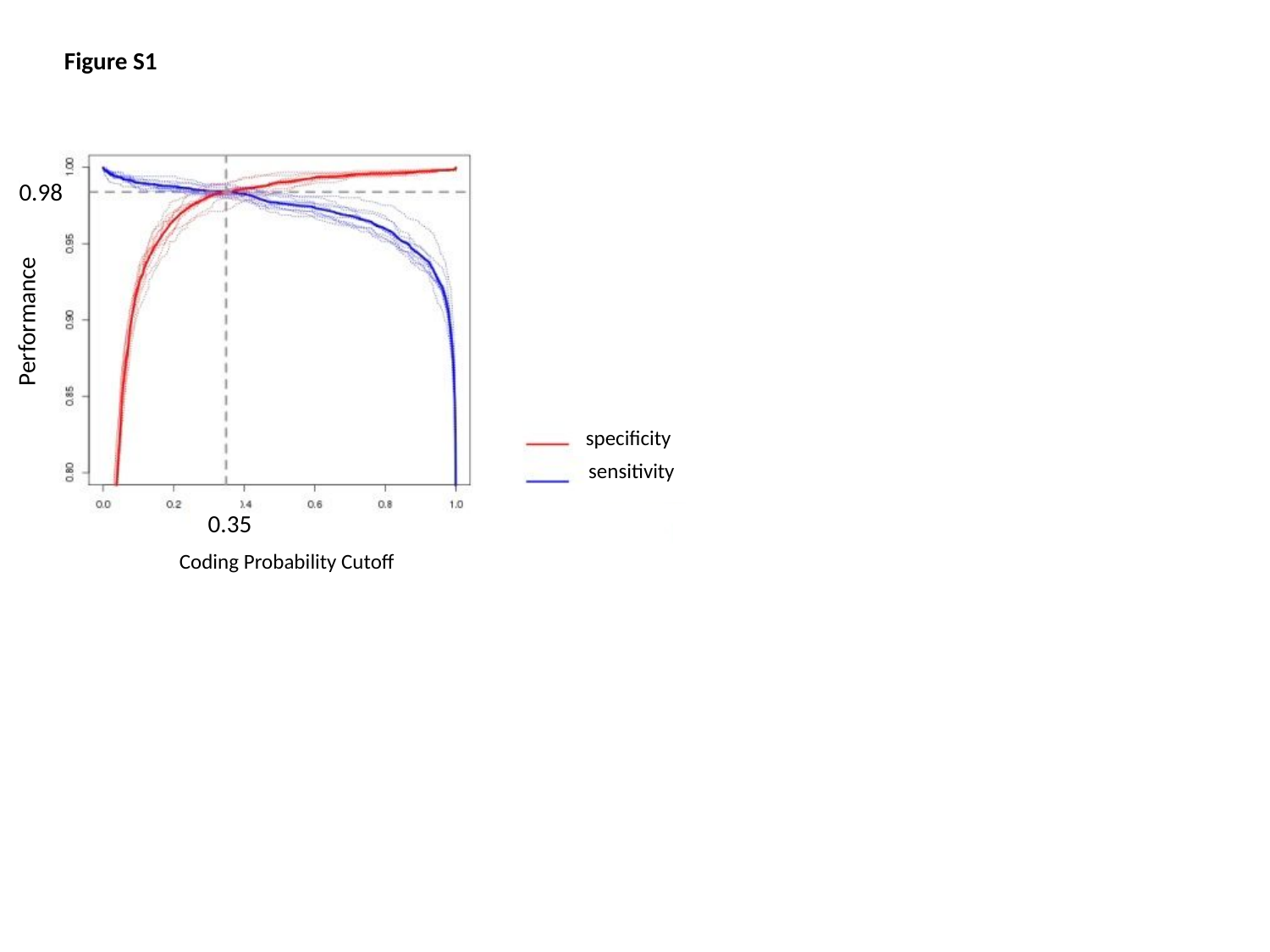

Figure S1
0.98
Performance
specificity
sensitivity
0.35
Coding Probability Cutoff

Supplement: Supplementary file 1 — Additional file 1: Figure S1: Performance evaluation using 10-fold cross-validation. (PPTX 75 KB) [file 12864_2014_6167_MOESM1_ESM.pptx]
