# Supplementary material for: Identification of large intergenic non-coding RNAs in bovine muscle using next-generation transcriptomic sequencing
Source: BMC Genomics. 2014 Jun 19;15(1):499. doi: 10.1186/1471-2164-15-499 (PMC4073507; doi:10.1186/1471-2164-15-499)
Supplement: Supplementary file 4 — Additional file 4: Figure S2: Distribution of lincRNA genes over all bovine chromosomes. (DOCX 12 KB) [file 12864_2014_6167_MOESM4_ESM.docx]

**Figure S2**

Count

Chromosome
