# Supplementary material for: Identification of large intergenic non-coding RNAs in bovine muscle using next-generation transcriptomic sequencing
Source: BMC Genomics. 2014 Jun 19;15(1):499. doi: 10.1186/1471-2164-15-499 (PMC4073507; doi:10.1186/1471-2164-15-499)
Supplement: Supplementary file 8 — Additional file 8: Table S6: Primer sequences used for RT-PCR. (DOCX 12 KB) [file 12864_2014_6167_MOESM8_ESM.docx]

**Table S6**

Number LincRNA name Forward primer sequence Reverse primer sequence Amplicon size (bp)

1 XLOC_018374 ATCAGGAAGGAGGAGGGTGT AGACCTACCCAGCGTCTTCA 500
 2 XLOC_000281 GTTAGCTGGGAACCAGTGGA CCCAAAGAGAATTCCGTCAA 491
 3 XLOC_026329 GTCCGCAGGTTGACCTAAAG GTTCATGCTTGGTGTGTGCT 501
 4 XLOC_010213 GCATCAGACCAACTTTCTCCA AAAAGCCGCATCAGGATACA 511
 5 XLOC_015677 AAAGGGCTGTGTCTGAATGG TTGCCTCTGTCATGTTGTCC 501
 6 XLOC_012543 GGGTTTGGGGGTAAGTAGGA TGCCTCTCTGTCCTCGAGTT 500
 7 XLOC_029392 ACACAGGCAGCAGAGAGGAT CCAGGCACTGTAACCATGAC 513
 8 XLOC_019164 GGTGGGTCTTGTCCTCTCTG CCCCTCGTGCAAACTTCTAA 501
 9 XLOC_016660 GCTGAGCAGAGGAGGCTAAA CTCCTTTCCTCCCTGAGAGC 499
 10 XLOC_029293 GAGGGCAGGTCGAGTCTCT ACGGAAGCCCTGAACACTTA 191
 11 XLOC_026330 TGAGGGCAGTGCTGTGTTAC AAGATGGGAGCCATTTTGTG 504
 12 XLOC_021462 AACAGTTCTGCCAGGGGAGT ACACACCGCATTGGAAGAG 394
 13 XLOC_011428 GAAAATTCCCTTGCCCTTTC GTCCCAAGAGGGAGGGATAG 492
 14 XLOC_002357 TGAGTTCATGGATGGCCTTT TGGCAAGTGGATTTTCCTTC 499
